# Increased levels of S100A8/A9, IL-1ß and IL-18 as a novel biomarker for recurrent tonsillitis

**DOI:** 10.1186/s12950-021-00290-8

**Published:** 2021-06-29

**Authors:** Christoph Spiekermann, Alicia Seethaler, Annika McNally, Markus Stenner, Claudia Rudack, Johannes Roth, Thomas Vogl

**Affiliations:** 1 Institute of Immunology, University Hospital Münster, Münster, Germany; 2 Department of Otorhinolaryngology, Head and Neck Surgery, University Hospital Münster, Kardinal-von-Galen-Ring 10, 48149 Münster, Germany

**Keywords:** Diagnosis, Recurrent tonsillitis, Interleukin, Calprotectin, Biomarker

## Abstract

**Background:**

Acute tonsillitis represents one of the most frequent reasons patients seek primary medical care and otorhinolaryngology consultation. Therefore, recurrent episodes of acute tonsillitis (RAT), also called chronic tonsillitis, exhaust a substantial amount of medical and financial resources. Diagnosis of tonsillitis depends on a physical examination, which therefore does not allow for a reliable differentiation between viral and bacterial infection. However, the frequency of bacterial infections during the previous three years is currently being used as the major deciding factor in patient selection for tonsillectomy. The aim of the present study was to determine an objective biomarker to help in the identification of patients suffering from recurrent tonsillitis.

**Results:**

By analyzing a panel of cytokines and chemokines in serum and saliva of patients with RAT compared to healthy controls, increased levels of IL-1ß (153.7 ± 48.5 pg/ml vs 23.3 ± 6.6 pg/ml, *p* = 0.021), IL-18 (120.2 ± 16.5 vs 50.6 ± 9.3 pg/ml, *p* = 0.007) and/or S100A8/A9 (996 ± 102 ng/ml vs 546 ± 86 ng/ml, *p* = 0.042) could be observed in patients suffering from RAT. Cut-off values of these parameters were determined and combined to a new RAT-score allowing for reliable identification of patients suffering from recurrent tonsillitis with a sensitivity of 95% and a specificity of 88%.

**Conclusion:**

The RAT-score represents the first objective criterion as a tool for the diagnosis of recurrent tonsillitis and it also improves patient selection for tonsillectomy.

## Background

As a part of the Waldeyer’s ring, the palatine tonsils represent secondary lymphoid organs and belong to the mucosa-associated lymphoid tissue (MALT) [1]. Although palatine tonsils show some similarities to the spleen, lymph nodes or Peyer’s patches of the gut, some unique characteristics exist. These include a partial capsule, the existence of lymphoreticular structures and an absence of afferent lymphatics [2]. The palatine tonsils represent the first line of defense against oropharyngeal pathogens and several reports have described their immunological significance [3–6]. In some cases, however, the palatine tonsils provoke increased inflammatory reactions associated with symptoms of a sore throat and a general malaise. Acute tonsillitis is the most common reason for emergency admission to otolaryngology service, recurrence of acute tonsillitis (RAT) and sore throat episodes in general, requiring a substantial utilization of medical resources [7, 8]. The etiology for the recurrence of acute tonsillitis in some patients still remains unclear. Tonsillectomy and tonsillotomy are very common procedures and several reports have described the benefits of these procedures in relation to recurrent tonsillitis and recurrent sore throat episodes [8–11]. However, tonsillectomy has become a source of controversy over the last years due to possible side effects, such as pain or postoperative haemorrhage, and the uncertainty of postoperative persistence of sore throat episodes [10, 12]. Currently, the diagnosis of RAT is based on subjective clinical signs and history taking and therefore objective criteria are still missing.

Inflammatory processes are initiated by the release of various cytokines and chemokines. The important influence of major mediators, such as TNF-α, IL-1ß and IL-6, on inflammatory processes is well reported. However, the role of other cytokines and chemokines, such as IL-12p70, IL-17A, and IL-33, as well as other soluble proteins, like the danger-associated molecular pattern S100A8/A9, have become of an increasing interest over the last years [13–16]. The heterodimeric complex S100A8/A9, also known as calprotectin, consists of the proteins S100A8 and S100A9 [17, 18]. S100A8/A9 is primarily expressed in neutrophil granulocytes and monocytes, but can also be observed in keratinocytes [19–22]. Several reports have described S100A8/A9 as a potential biomarker in different chronic and acute diseases, including diseases of the oropharynx. We have recently shown that S100A8/A9 in combination with characteristic symptoms serves as a biomarker in a peritonsillar abscess [23]. The broad range of cytokines, chemokines and DAMPs being involved in inflammatory processes led to the assumption that they could serve as helpful biomarkers in identifying patients with recurrent tonsillitis. It was the aim of the present study to develop an objective biomarker to help identify patients with potential recurrent tonsillitis and to thereby improve the patient selection for tonsillectomy.

## Results

### Cytokines and Chemokines

**Fig. 1:**
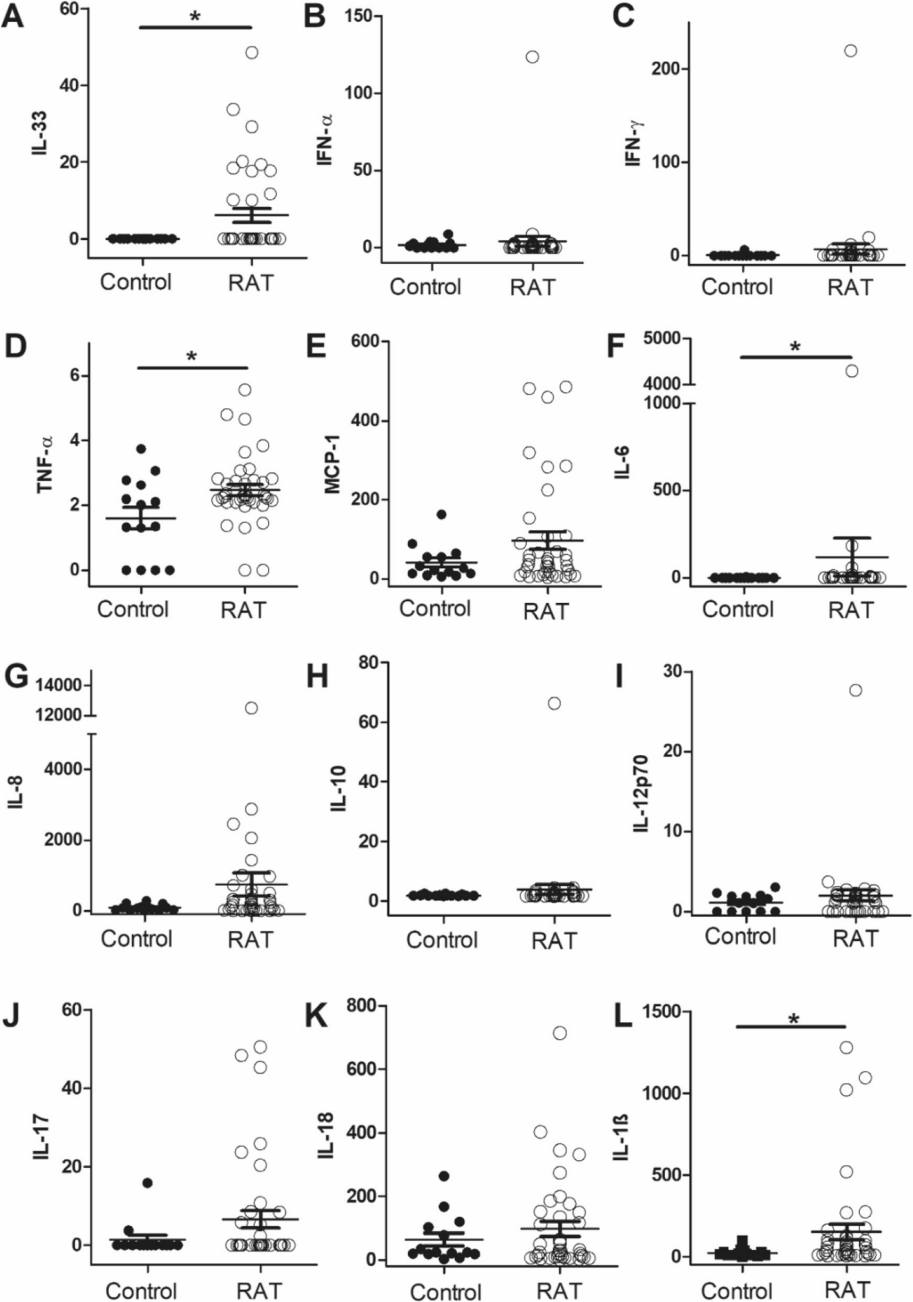
Cytokine levels of IL-33 (**A**), IFN-α (**B**), IFN-γ (**C**), TNF-α (**D**), MCP-1 (**E**), IL-6 (**F**), IL-8 (**G**), IL-10 (**H**), IL-12p70 (**I**), IL-17 (**J**), IL-18 (**K**) and IL-1ß (**L**) in saliva. Levels of IL-1ß, IL-6, TNF-α and IL-33 are significantly increased in patients with recurrent tonsillitis (RAT) compared to healthy controls (*p < 0.05). All values are given in [pg/ml]

After quantifying the salivary levels of IL-8, IL-10, IL-12p70, IL-17A, IL-18, IFN-α, IFN-γ and MCP-1 in patients suffering from RAT, we could not observe significant differences compared to controls. Although salivary levels of IL-6, IL-33 and TNF-α were significantly increased in patients with recurrent tonsillitis compared to healthy controls, these parameters were unable to differentiate between the diagnosis groups since significance was only achieved through single outliers (Fig. 1 A–K). ROC analysis for IL-6, IL-33 and TNF-α revealed *A*-values <0.7 and confirmed their ineligibility as a potential biomarker (data not shown). Only the significantly increased levels of IL-1ß had the potential to serve as an appropriate biomarker for recurrent tonsillitis (153.7 ± 48.5 pg/ml vs 23.3 ± 6.6 pg/ml, *p* = 0.021) (Fig. 1 L). A cut-off value of 30pg/ml was determined by ROC analysis to identify patients suffering from recurrent tonsillitis with a sensitivity of 0.69 and a specificity of 0.88.

**Fig. 2:**
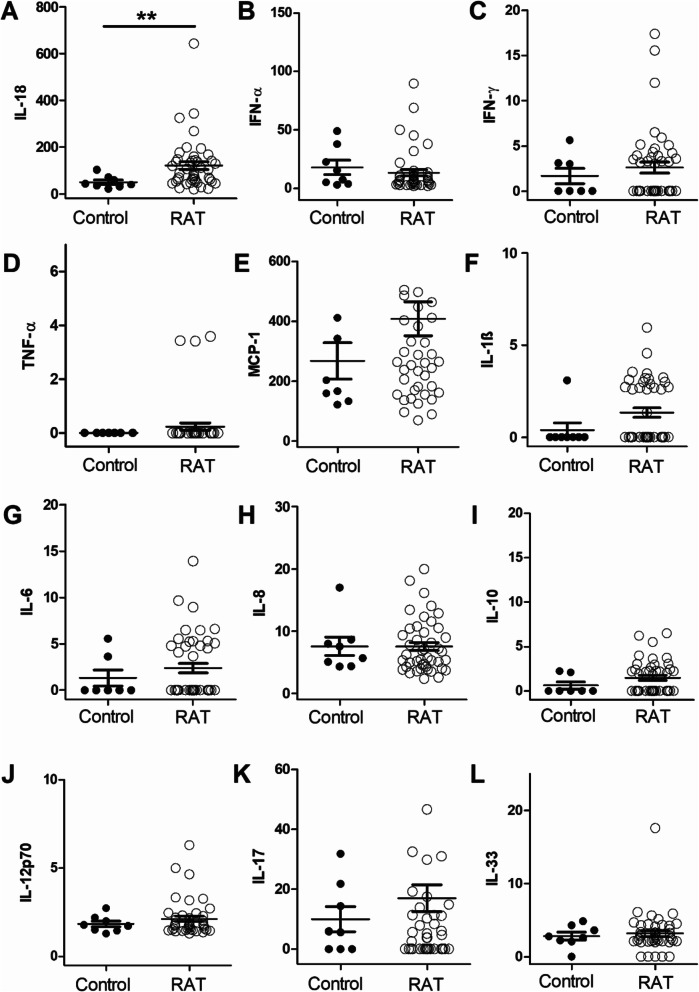
Cytokine levels of IL-18 (**A**), IFN-α (**B**), IFN-γ (**C**), TNF-α (**D**), MCP-1 (**E**), IL-1ß (**F**), IL-6 (**G**), IL-8 (**H**), IL-10 (**I**), IL-12p70 (**J**), IL-17 (**K**) and IL-33 (**L**) in serum. Significantly increased levels of IL-18 could be observed in patients with recurrent tonsillitis (RAT) compared to healthy controls (**p < 0.01). All values are given in [pg/ml]

In serum, significantly increased levels of IL-18 could be observed in patients with RAT compared to controls (120.2 ± 16.5 vs 50.6 ± 9.3 pg/ml, *p* = 0.007) (Fig. 2 A). IL-18 showed a sensitivity of 0.94 and a specificity of 0.63 in the differentiation between healthy controls and patients suffering from RAT (cut-off: 44 pg/ml). Levels of IFN-α, IFN-γ, TNF-α, IL-6, IL-8, IL-10, IL-12p70, IL-17A, IL-33 and MCP-1 showed no significant differences between the healthy and the RAT group (*p* ≥ 0.05) (Fig. 2 B–L).

### S100A8/A9

**Fig. 3:**
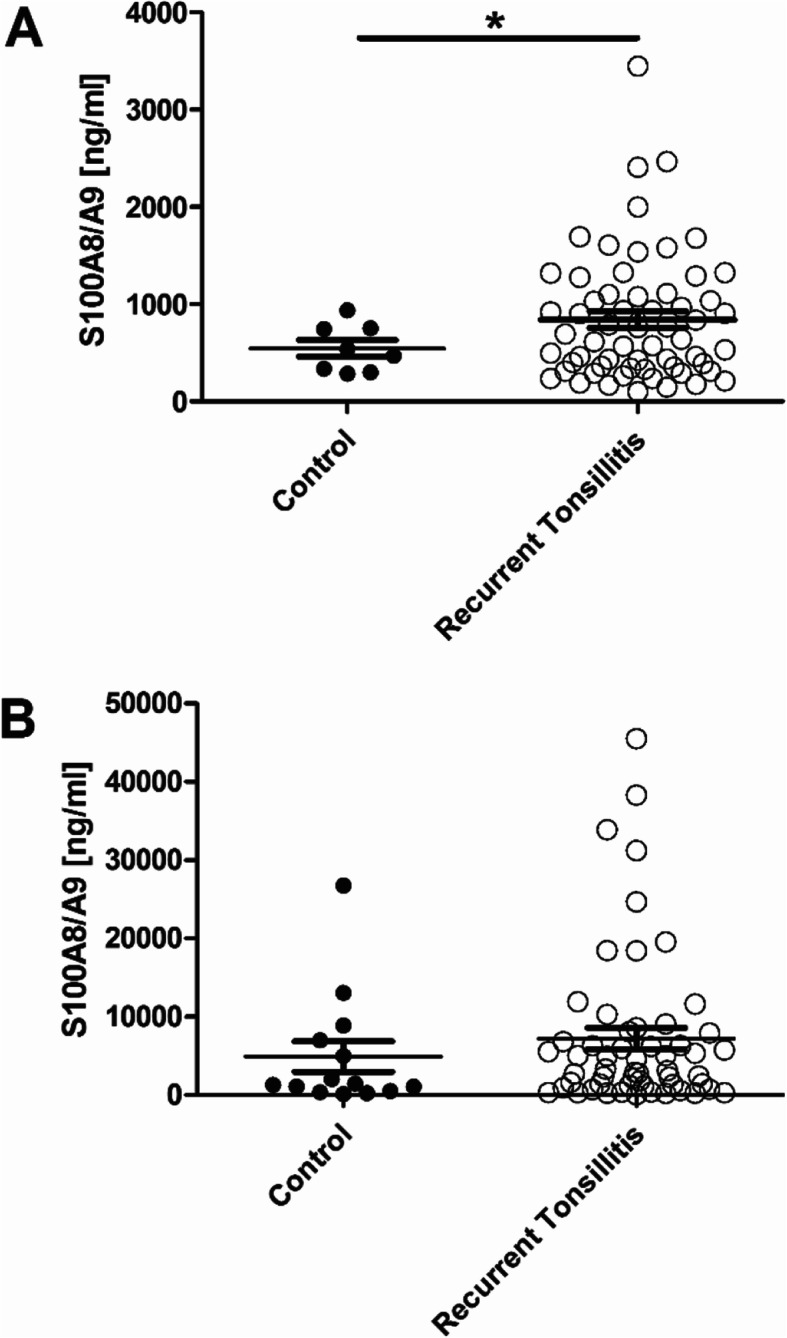
S100A8/A9 levels in serum (**A**) and saliva (**B**) of patients with recurrent tonsillitis (RAT) compared to healthy controls (*p < 0.05)

Significantly increased levels of S100A8/A9 could be observed in the serum of RAT patients (996 ± 102 ng/ml vs 546 ± 86 ng/ml, *p* = 0.042) (Fig. 3 A) compared to the controls. However, this could not be observed in the saliva of patients with RAT when compared to the controls (8666 ± 1636 ng/ml vs 4936 ± 1976 ng/ml, *p* = 0.072) (Fig. 3 B). ROC analysis of S100A8/A9 levels in serum revealed a 757 ng/ml cut-off value that could be used to distinguish between RAT and healthy controls with a sensitivity of 60% and a specificity of 88%.

### Development of the RAT-Score

**Fig. 4:**
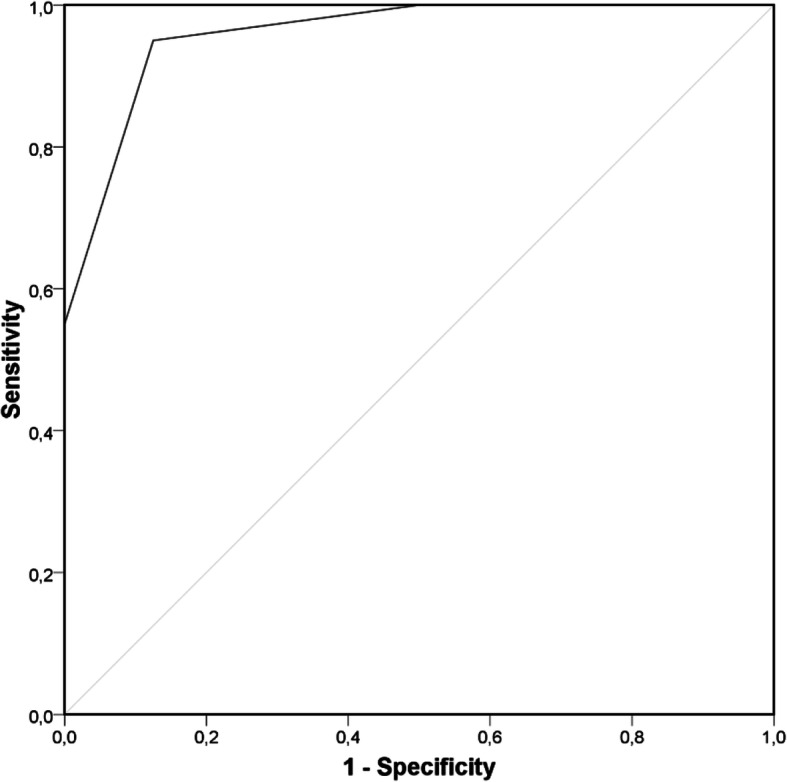
Receiver Operating Characteristic curve analysis of the RAT score. A cut-off value of 1.5 could be determined for the RAT score in order to identify patients with recurrent tonsillitis with a sensitivity of 0.95 and a specificity of 0.88. Area under the curve *A* = 0.959

In order to establish an objective measure for a reliable identification of patients with recurrent tonsillitis, a high sensitivity and specificity is required. Therefore, the RAT-score was created as a scoring method based upon the cytokines and soluble proteins that have demonstrated their potential to discriminate between RAT and controls through their significant increased levels in saliva or serum, as well as their corresponding ROC analysis. One point is added to the RAT-score for each parameter exceeding the cut-off value of IL-1ß in saliva (30 pg/ml), IL-18 (44 pg/ml), and/or S100A8/A9 (757 ng/ml) in serum (Table 1). ROC analysis revealed a cut-off value of 1.5 for the existence of RAT with a sensitivity of 95% and a specificity of 88% (Fig. 4).

**Table 1:** RAT-score. One point is added for each elevated parameter above the cut-off to determine the RAT-score. Values ≥2 indicate the diagnosis of recurrent tonsillitis

		Cut-off	Sensitivity	Specificity	Points
S100A8/A9	Serum	757 ng/ml	0.60	0.88	1
IL-1ß	Saliva	30 pg/ml	0.69	0.88	1
IL-18	Serum	44 pg/ml	0.94	0.63	1
**RAT-score**		**1.5 points**	**0.95**	**0.88**	**0-3**

### Quality of Life

The outcome of tonsillectomy was assessed within a small group of patients (n = 9) with a median age of 23 years (range, 16-28) and a RAT score of 2.4 ± 0.5 (mean ± SD). Preoperatively, the patients reported a high restriction of the quality of life due to recurrent episodes of tonsillitis with a TOI-14 score of 42.8 ± 13.9 (mean ± SD). A significant reduction in score could be achieved through a tonsillectomy, resulting in a TOI-14 score of 10.7 ± 11.9 (mean ± SD, *p* < 0.001) reported at a median of 23 months (range, 16-28) following the procedure. These findings indicate a lower perceived burden of disease by the patients.

## Discussion

Although various reports have been focused on the expression of cytokines or chemokines in the course of recurrent tonsillitis, this is the first report about an objective criterion that can be used to identify patients suffering from recurrent tonsillitis.

By recommendation of Paradise et al, as well as other guidelines, the indication for tonsillectomy or tonsillotomy depends on the frequency of bacterial tonsillitis episodes during the previous three years [24–26]. This recommendation may be associated with several biases and disadvantages. The diagnosis of tonsillitis is based on physical examination which does not allow for a reliable differentiation between bacterial and viral infection [7, 10, 12]. Due to the high percentage of acute tonsillitis being of viral etiology (50-80% of the cases) and to the frequency of antibiotics prescription, it could be assumed that a high percentage of diagnoses are incorrect [7, 27]. In this context the limited value of the Centor criteria should be mentioned. Briefly, one point each is added for symptoms like fever, swollen cervical lymph nodes, absence of cough and tonsillar exudates [28]. The Centor criteria only provide an estimation of the probability of an infection with *Group A* streptococcus, but does not provide a precise differentiation between bacterial and viral infection [29, 30]. Furthermore, a swab of the exudative tonsillitis to determine its etiology is considered inappropriate since most pathogens belong to the healthy flora and positive results for tonsillitis is seen in 40% of asymptomatic carriers [7, 12]. Inconsistent medical documentation or incorrect information from the patients causes a bias in taking medical history with an over- or underestimation of the frequency of bacterial tonsillitis episodes [9]. Due to the vague statements of the patients, the variability in diagnosis of bacterial tonsillitis and the associated doubtful use of antibiotic treatment, the frequency of episodes of bacterial tonsillitis seems to be an ineligible criterion for identification of patients who may benefit from tonsillectomy. Despite this uncertain selection of patients, several reports have shown the benefits of the tonsillectomy. Patients who previously suffered from recurrent tonsillitis or sore throat showed a significant decrease in sore throat episodes, missed-work or school days, a reduction in the use of medical resources and an increase in quality of life [8, 9, 11, 31, 32]. These benefits need to be weighed against the possible risks of surgery, such as postoperative hemorrhage, general morbidity or a prolonged hospitalization. This calls for the need of a novel, objective criterion or diagnostic test allowing for an improved, specified patient selection. The presented RAT-score is the first report of an instrument enabling the objective identification of patients suffering from recurrent tonsillitis.

The new scoring system proposed in this manuscript does not allow for a distinction between bacterial and viral tonsillitis. However, this was not the aim of the present study. The aim of our study was to determine an objective set of biomarkers to identify patients who would benefit from tonsillectomy regardless of the number of previous tonsillitis episodes and regardless of whether the tonsillitis was of bacterial or viral origin. Hence, the RAT score identifies cases benefiting from tonsillectomy which would be missed, if a decision for tonsillectomy was based solely on the recommendations of the current guidelines.

Tonsils are covered by a stratified squamous non-keratinized epithelium [33]. The tonsillar epithelium is characterized by crypts extend across the epithelial surface. It is also described as a specialized lymphoepithelium that contains a high amount of lymphocytes, as well as macrophages and dendritic cells found in the tonsil’s stroma [2, 34, 35]. As part of the MALT, the palatine tonsils play a pivotal role in the identification of pathogens and the presentation of antigens in order to generate effector and memory cells of the adaptive immunity [35]. These processes are well regulated by various cytokines, chemokines and soluble proteins [35]. Recent work focused on cytokines concerning the activation and regulation of the adaptive immune response; however, the role of the innate immunity in recurrent tonsillitis was rather neglected [36–38].

Recently we could show increased levels of S100A8/A9 in the saliva and serum of patients suffering from peritonsillar abscess. We were also able to observe S100A8/A9 to be primarily expressed in the tonsillar epithelium of tonsils [23]. Furthermore, S100A8/A9 was found to be expressed in gingival epithelial cells, particularly in differentiated keratinocytes, and elevated levels could be observed in cases of periodontal and oral inflammatory diseases [19, 39, 40]. We proposed an influence of this S100A8/A9 complex on the pathomechanism of recurrent tonsillitis and could also observe elevated levels of this complex in serum of patients with recurrent episodes of acute tonsillitis. Although antimicrobial properties of S100A8/A9 and S100A8/A9-dependent resistance of oral mucosal keratinocytes to bacterial invasion have been described, the role of this complex in mucosal and particularly in tonsillar immunology is still unclear [19, 20].

Interestingly, we could show increased levels of IL-1ß and IL-18 in patients with RAT. These cytokines both belong to the IL-1 family and are secreted in a caspase-1 dependent manner [41]. Furthermore, increased levels of IL-1ß and IL-18 in epidermal keratinocytes are associated with the inflammasome NLRP-3 activity, which is further activated by S100A8/A9 [42, 43]. Hence, a common pathway and a pathogenic association between these increased levels of S100A8/A9, IL-1ß and IL-18 in tonsillar epithelium is plausible. Remarkably, single outliers were observed in the determination of the cytokine levels. However, these outliers were found in different patients and even after removing these outliers, the differences of IL-1ß as well as IL-18 and S100A8/A9 concentrations between the RAT and the control group were still significant.

Using ROC analysis, we determined the potential of each cytokine to be used as a biomarker. An *area under the curve*–value (*A*-value) above 0.7 was crucial for this distinction. We used ROC curve analysis to determine cut-off values, because this method is considered to be less susceptible for being distorted by outliers than using the mean. Since this is the first report of the above mentioned cytokines to be elevated in patients with RAT, we decided to include the data unfiltered.

We were able to show that all patients with a RAT-score value ≥2 benefited from tonsillectomy, which was indicated by a significant reduction in the TOI-14 score postoperatively. It should be noted that the data was acquired within a small group of patients and is thus considered to be preliminary.

Further retrospective and prospective, randomized studies should be performed to analyze the capacity of the RAT score as a predictor concerning the recurrence of tonsillitis or sore throat episodes and the outcome of tonsillectomy. Larger cohorts are necessary to confirm or refine the proposed cut-off values. We will continue to examine the influence of S100A8/A9 on the pathomechanism of recurrent tonsillitis and its function in healthy tonsils. In addition, we will also examine the relevant pathways in order to explain the association of increased levels of IL-1ß, IL-18 and S100A8/A9 in patients suffering from recurrent tonsillitis.

## Conclusion

Elevated levels of IL-18, S100A8/A9 specifically in serum and IL-1β in saliva can be used as a novel, objective biomarkers and collectively scored using the RAT-score to identify patients with recurrent tonsillitis. Increased levels of at least two out of the three biomarkers will positively identify such a patient. During patient selection for tonsillectomy, the RAT-score can be used as a guideline to allow for better and more appropriate patient selection.

## Methods

### Study Population

Patients (N = 71) in the study cohort were diagnosed with recurrent tonsillitis, enrolled, and underwent tonsillectomy during an asymptomatic interval without acute inflammation. The study population with a median age of 24 years (range, 13-59 years) consisted of 33 male and 38 female patients (male-to-female ratio, 0.86:1). These patients reported 11.6 ± 5.9 (mean ± SD) episodes of recurrent tonsillitis in the three years preceding this study. Healthy volunteers who had no past history of recurrent tonsillitis, previous tonsillectomy or tonsillotomy (n = 15; male-to-female ratio, 0.67:1) served as controls. The control subjects had a median age of 30 years (range, 26-59 years).

### Ethical approval

This study was performed in the Department of Otorhinolaryngology, Head and Neck Surgery, University Hospital Münster in accordance with ethical principles, including the World Medical Association Declaration of Helsinki (version 2002) and the additional requirements, and has been approved by the institutional ethics committee [2015-217-f-S]. Written informed consent was obtained from all subjects.

### Acquisition of sera and saliva samples

To isolate the serum faction, blood was allowed to clot and later centrifuged at 2000g for 10 minutes within 2 hours after acquisition. Saliva acquisition was performed with untreated Salivette® (Sarstedt, 51.1534) as described in the manufacturer’s datasheet or through collection of saliva in a 50ml Falcon tube and centrifugation at 1000g for 15 minutes. Samples were immediately processed after acquisition. Supernatants were aliquoted and stored at -20 °C until analysis. Generally, saliva and serum samples were taken at the time point of study enrollment. This occurred at the same time as consent was given for surgery several days prior to surgery. It was crucial that the patients were symptom-free at the time point of sample acquisition.

### Analysis of chemokines, cytokines and soluble proteins

Quantification of cytokines/chemokines in serum and saliva was performed with the LEGENDplex^TM^ assay “Human Inflammation Panel” (BioLegend) as described in the manufacturer’s manual. The “Human Inflammation Panel” allows simultaneous quantification of IL-1ß, IL-6, IL-8, IL-10, IL-12p70, IL-17A, IL-18, IL-33, IFN-α, IFN-γ, MCP-1 and TNF-α. Fluorescent signal intensities were detected by NAVIOS^TM^ Flow Cytometer (Beckmann Coulter). S100A8/A9 concentrations were measured with a sandwich enzyme-linked immunosorbent assay (ELISA) for human S100A8/A9 as described earlier [21].

### Quality of Life in patients with recurrent tonsillitis

Quality of life was assessed pre- and postoperatively by the tonsillectomy outcome inventory 14 (TOI-14) which represents a disease-specific questionnaire for patients with recurrent tonsillitis. The TOI-14 is a validated and reliable instrument consisting of 14 questions concerning throat discomfort, general health, use of resources and social/psychological restrictions. The questions are answered using a six-point Likert scale. A score from zero to 100 points can be calculated, where higher scores indicate a higher burden of disease [44].

### Statistical Analysis

Statistical analyses were performed with IBM® SPSS® Statistics 24 and SigmaPlot®12. Results are described as mean values ± standard error of the mean (mean ± SEM) or mean value ± standard deviation (mean ± SD), as indicated in the figures. Correlations between variables were determined by Pearson’s correlation coefficients (r_p_) and were considered to be low (0.2 < r_p_ ≤ 0.5), good (0.5 < r_p_ ≤ 0.8) or excellent (0.8 < r_p_ ≤ 1.0). Student t-test was used to detect significant differences in parametric results and Mann-Whitney U test was performed to analyze differences between non-parametric groups. Furthermore, Kruskal-Wallis test was used to reveal differences between more than two non-parametric groups. *P*-values ≥0.05 are considered not to be significant. Significant results are marked with asterisks (*p < 0.05, **p < 0.01, ***p < 0.001). The capacity of the model to differentiate between positive and negative results is described by area under the curve values (*A*-values) and illustrated by ROC curves which allow calculation of cut-off values. Discriminative power of the model is considered to be excellent with an *A*-value of ≥0.9, good ≥0.8, and acceptable ≥0.7. *A-*values <0.7 were considered not to be sufficient for a potential biomarker.

## Data Availability

The datasets used and/or analyzed during the current study are available from the corresponding author on reasonable request.
